# Quality control of VMAT synchronization using portal imaging

**DOI:** 10.1120/jacmp.v16i1.5238

**Published:** 2015-01-08

**Authors:** James L. Bedford, Honorata Chajecka‐Szczygielska, Michael D. R. Thomas

**Affiliations:** ^1^ Joint Department of Physics at The Institute of Cancer Research and The Royal Marsden NHS Foundation Trust London UK

**Keywords:** quality control, VMAT, IMRT, linear accelerator, portal image

## Abstract

For accurate delivery of volumetric‐modulated arc therapy (VMAT), the gantry position should be synchronized with the multileaf collimator (MLC) leaf positions and the dose rate. This study, therefore, aims to implement quality control (QC) of VMAT synchronization, with as few arcs as possible and with minimal data handling time, using portal imaging. A steel bar of diameter 12 mm is accurately positioned in the G–T direction, 80 mm laterally from the isocenter. An arc prescription irradiates the bar with a 16mm×220mm field during a complete 360° arc, so as to cast a shadow of the bar onto the portal imager. This results in a sinusoidal sweep of the field and shadow across the portal imager and back. The method is evaluated by simulating gantry position errors of 1°–9° at one control point, dose errors of 2 monitor units to 20 monitor units (MU) at one control point (0.3%–3% overall), and MLC leaf position errors of 1 mm ‐ 6 mm at one control point. Inhomogeneity metrics are defined to characterize the synchronization of all leaves and of individual leaves with respect to the complete set. Typical behavior is also investigated for three models of accelerator. In the absence of simulated errors, the integrated images show uniformity, and with simulated delivery errors, irregular patterns appear. The inhomogeneity metrics increase by 67% due to a 4° gantry position error, 33% due to an 8 MU (1.25%) dose error, and 70% due to a 2 mm MLC leaf position error. The method is more sensitive to errors at gantry angle 90°/270° than at 0°/180° due to the geometry of the test. This method provides fast and effective VMAT QC suitable for inclusion in a monthly accelerator QC program. The test is able to detect errors in the delivery of individual control points, with the possibility of using movie images to further investigate suspicious image features.

PACS numbers: 87.55.Qr, 87.56.bd, 87.56.Fc

## I. INTRODUCTION

Clinical use of volumetric‐modulated arc therapy (VMAT)^1^, ^2^, ^3^, ^4^ brings with it the requirement for regular quality control (QC) of arc delivery on the linear accelerator. Such quality control gives confidence that the accelerator can reliably deliver VMAT prescriptions. While pretreatment verification may serve to ensure that a particular accelerator delivers a particular prescription accurately,^(5)^ it is often necessary to transfer a patient's treatment to another accelerator during the course of treatment and, in this case, it is important to be confident that the replacement accelerator can deliver the prescription accurately, without reverifying the prescription.

The specific tests required for QC of VMAT are now well‐established,^6^, ^7^, ^8^ and a proposal has been made for the frequency of these tests.^(7)^ Broadly, there are three types of test needed: 1) measurement of beam flatness and symmetry at the range of dose rates used for VMAT; 2) demonstration of accurate multileaf collimator (MLC) calibration;^9^, ^10^, ^11^, ^12^, ^13^, ^14^, ^15^, ^16^ and 3) ensuring accurate synchronization of gantry, MLC, and dose rate during VMAT delivery.^(17)^ Carrying out these tests explicitly represents a significant workload during monthly accelerator QC. A mitigating factor is that test type 1) and 2) are very similar to tests that are normally carried out for conformal and intensity‐modulated radiation therapy (IMRT).^(18)^ These items can, therefore, be merged with the standard QC procedure already in place in a typical department. However, the final type of test, that of delivery synchronization, remains an important and substantial item.

It may be possible to use the dynamic log files of the accelerator to verify that synchronization is correct (see Schreibmann et al.^(5)^ for an example in the patient‐specific context), but a method independent of the accelerator is desirable. Portal imaging may be used for this purpose (e.g., Liu et al.,^(19)^ again in the pretreatment context). Commercial array phantoms are designed primarily for patient‐specific quality assurance rather than accelerator quality assurance, but may be used successfully to test synchronization.^(17)^ The general approach for machine‐specific quality assurance of synchronization is to use a prescription which produces a dose distribution sensitive to the delivery parameters.^20^, ^21^ A method previously proposed for testing delivery synchronization^(7)^ irradiates a transaxial film in a cylindrical phantom with a narrow aperture, the aperture being designed to move in a sinusoidal pattern around an offset isocenter. However, this suffers from two drawbacks: firstly, the use of a transaxial film in a cylindrical phantom with the collimator at 0° actually only tests the synchronization of one MLC leaf pair; and secondly, it requires the use of radiochromic film, which is increasingly unpopular for radiation therapy dosimetry. A method testing all MLC leaf pairs and using a portal imager would therefore be considerably more attractive. The optimal VMAT QC procedure should be both simple and efficient to execute and use the portal imager, whilst testing all of the MLC leaves simultaneously. This paper proposes such a method, with the aim of providing a clear, easily visible indication of synchronization errors using minimal equipment and a simple setup. This paper investigates the sensitivity of the method to potential errors and gives some representative results following its implementation into clinical use.

## II. MATERIALS AND METHODS

### A. Test procedure

**Figure 1:**
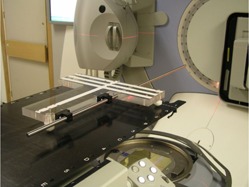
Apparatus for accurately supporting the off‐axis steel bar for VMAT QC (and for laser QC measurements).

The synchronization test is carried out by positioning a 12 mm diameter steel rod longitudinally along the couch, 80 mm to one side of the isocenter. The couch is retracted so that it does not attenuate the beam in any way. In our implementation, the rod has been integrated with a PMMA laser QC board (Fig. 1). A counterclockwise VMAT arc is then delivered from gantry angle 179.9° to 180.1°, consisting of 37 control points at 10° intervals. The control points are merely used to synchronize the delivery and the success of the method does not depend on their position. If an error occurs between control points, synchronization is lost and the portal imager detects the result. Thus, the method can detect errors of much less than 10° and in between control points, as the results show. The method is therefore suitable for use with RapidArc (Varian Medical Systems, Palo Alto, CA) (2° control point spacing) or SmartArc (Philips Radiation Oncology Systems, Madison, WI) (2°, 3°, 4°, or 6° control point spacing). The method is also general enough to be applicable to any accelerator in common clinical use. The beam aperture is 220mm×16mm, directed by means of a sinusoidal pattern to coincide with the steel bar. The beam prescription is produced using in‐house software and is in the form of a DICOM file, which is widely applicable.

**Figure 2:**
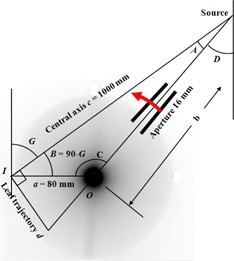
Geometrical construction for determining aperture position for the synchronization test. The grayscale pattern indicates the approximate dose distribution that would be obtained if a phantom had been positioned on the couch in place of the steel bar.

Referring to Fig. 2, the objective is to find d, the off‐axis position of the center of the aperture (defined at the isocenter plane) for some gantry angle G at a control point such that, with the beam isocenter at I, the center of the aperture passes through O, the centerline of the steel bar. We require:

(1) $$d=c\text{tan}A=\pm c\sqrt{{\text{sec}}^{2}A-1}$$

By repeated application of the trigonometric identity

(2) $$\text{cos}C=\frac{a^{2}+b^{2}-c^{2}}{2ab}$$

it follows that:

(3) $$d=\pm c\sqrt{\frac{a^{2}+c^{2}-2ac\text{sin}G}{{\left(c-a\text{sin}G\right)}^{2}}-1}$$

where the sign depends upon which quadrant the gantry angle is in. Eq. (3) is used to determine the position of the midline between the two leaf banks at each control point. The offset of the two MLC leaf banks from this midline is then chosen so that the leaves remain a constant distance from the steel bar. First note that the plane, orthogonal to the beam axis, which contains the center of the steel bar, is a distance c–acosB=c–asinG from the source. Then if r is the radius of the steel bar plus a small margin, it follows from similar triangles that the divergent projection of r onto the isocenter plane is:

(4) $$w=\pm \frac{c}{c-a\text{sin}G}\cdot r$$

which is the desired leaf position. The radius, r, is set in this study to be 8 mm, based on empirical tests which show that an additional 2 mm over the 6 mm radius of the bar is optimal for showing the position of the MLC leaves on cine images. Application of these formulae to each control point gives the overall prescription to be delivered.

The monitor units are chosen, based on previous studies,^(7)^ such that the total for the complete 360° arc is 640 MU. These monitor units give dose rates comparable to those used by typical clinical plans (i.e., between 100 and 600 MU/min). Since the aperture moves in a sinusoidal pattern, the leaves spend more time at the extrema of motion than at the central axis. Therefore, if the dose rate were constant, a greater fluence would be delivered to the imager at the extrema than at the central axis. Hence, in this study, the number of monitor units delivered between each pair of control points is proportional to the distance travelled by the center of the aperture between those control points, so that the integrated fluence delivered to the portal imager is uniform. Consequently, the dose rate is lower at gantry angles 0° and 180°, where the aperture is at its extrema, than at gantry angles 90° and 270°, where the aperture is at the central axis. Other dose rates can be tested by changing the total monitor units.

For the bar to remain centered between the MLC leaves, the gantry position and MLC leaf position must be correctly synchronized. Moreover, the dose rate must also be synchronized to give the correct uniform intensity at the imager as the aperture moves across the imaging panel with varying speed. Thus, the gantry position, dose rate, and MLC leaf position must be correct for the test to succeed. Note that the preceding methods are independent of beam energy, so the test is suitable for use at a range of photon energies, for example from 4 MV to 20 MV. An energy of 6 MV is used throughout this study as 6 MV is used exclusively for VMAT treatments at our institution.

An iViewGT portal imager (Elekta AB, Stockholm, Sweden) is then used to measure an integrated image of the beam. The presence of the steel bar attenuates the beam slightly, so that the 2 mm wide region either side of the bar contributes to the portal image more than the region underneath the bar. Any MLC leaf inaccuracy is thereby accentuated. Due to the 260mm×260mm imaging size (at isocenter) of the portal imager, the test requires a counterclockwise arc and a clockwise arc to fully evaluate the VMAT performance of all MLC leaves. The test can conveniently be arranged so that half of the leaves are tested with the counterclockwise arc and the remaining half are tested with the clockwise arc.

In the event of any inaccuracy showing itself on the integrated images, the test can be repeated using movie portal images, so that the precise problem can be visualized. In this case, the steel bar can be clearly seen on the images, and should be symmetrically centered in the beam aperture at all control points.

### B. Sensitivity to errors

The above test was run using a Synergy accelerator with Agility head (Elekta AB, Stockholm, Sweden). The portal images were viewed as integrated images. The sensitivity of the method to errors was tested by simulating a gantry position error of between 1° and 9° at one control point, a dose error of between 2 and 20 MU (i.e., 0.3%–3% of overall dose) at one control point, and an MLC leaf position error of between 1 and 6 mm in eight leaves at one control point. All of these errors were at gantry angle 90° where the sensitivity of the method was greatest. To evaluate the impact of gantry angle on the sensitivity of the method to the various errors, the three types of error were introduced at gantry angles 0° to 90° in steps of 10°. The sensitivity of the method to MLC leaf position error was further investigated by varying the number of leaves affected by the error from 1 to 16. A static image of a large open field was also taken at gantry angle 0° to assist with the quantitative analysis of the images. To evaluate the images quantitatively, they were first registered for translation and rotation to the corresponding open image. The rest of the analysis was performed on the quotient of the two images divided by the overall median of the modulated image. Division by the median was necessary because the modulated field and the open field had different monitor units and the resulting images had different intensities. Therefore the quotient, while it removed the inherent intensity variation of the modulated image, was not necessarily unity in the absence of errors. Division by the median of the modulated image was, therefore, additionally performed.

**Figure 3:**
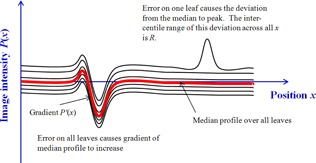
Illustration of $P_{95}^{\prime }$ and R statistics.

Midleaf profiles were extracted, consisting of those pixels in the central fifth of each leaf width. To avoid image‐edge effects, the profiles of the first and last leaves were displaced 1.5 to 2 mm inwards. Each profile was then normalized by subtracting its median value. The median value of all the normalized profiles was then calculated at each pixel across the width of the image in the direction of leaf motion, x, to give a single median profile, P(x). The absolute value of the spatial derivative, $P^{\prime }(x)$ of this median profile, was calculated and its 95th percentile value over all $x,P_{95}^{\prime }$, collected (Fig. 3). This statistic was sensitive to those errors which affected all leaves (gantry and dose in our tests).

To evaluate anomalous behavior in fewer than all the leaves, the variation from the median profile was obtained by subtracting the median profile from each leaf profile. The resulting difference profiles were then individually characterized by the difference, R, between their 5th and 95th percentiles. They were also collectively characterized by the maximum value of the R statistic, $R_{\text{max}}$, over all of the profiles (Fig. 3).

To evaluate the potential impact of the errors introduced into the prescriptions,^(22)^ the prescriptions were applied in a Pinnacle^3^ treatment planning system (v9.6; Philips Radiation Oncology Systems) to a cylindrical water phantom of diameter 200 mm, with the isocenter positioned 80 mm to one side. Dose was computed for each of the prescriptions on a 4mm×4mm×4mm grid using collapsed cone convolution. The prescriptions with errors applied were then compared with that having no error, using dose distributions and dose‐volume histograms for cylinders of 40 mm and 120 mm diameter, concentric with the central axis of the phantom, and with length 220 mm, equal to the length of the prescribed fields.

The prescriptions with deliberate errors at gantry 90° were also recalculated on a dataset representing a Delta^4^ phantom (ScandiDos, Uppsala, Sweden),^(23)^ using a dose grid of resolution 2mm×2mm×2mm and the resulting dose distributions were exported to the Delta^4^. The prescriptions were then delivered and the dose distributions measured with the Delta^4^ were compared with the planned dose distribution in the absence of errors and also with the measured dose distribution in the absence of errors. The first of these two comparisons was intended to represent the normal verification procedure and the second was intended to represent the impact of the delivery errors in isolation from the uncertainty associated with the planning system calculation. As with the portal image measurements, the isocenter was positioned 80 mm laterally to the central axis of the phantom. The results were corrected for the daily output of the accelerator. Comparisons were by means of a gamma index for 3% and 3 mm,^(24)^ with 100% defined as the dose at the center of the phantom. All measurements above 10% were included in the analysis, and 95% of measurements were required to have a gamma index of less than unity.

### C. Clinical application

The VMAT QC method was implemented on five Synergy accelerators, two with MLCi heads and three with Beam Modulator heads (Elekta AB). The two statistics described above, $P_{95}^{\prime }$ and R, were collected to assess stability and to select investigation levels for assessment of future performance. All of the accelerators had previously undergone full commissioning for VMAT using the procedure of Bedford and Warrington.^(7)^

## III. RESULTS

### A. Sensitivity to errors

**Figure 4:**
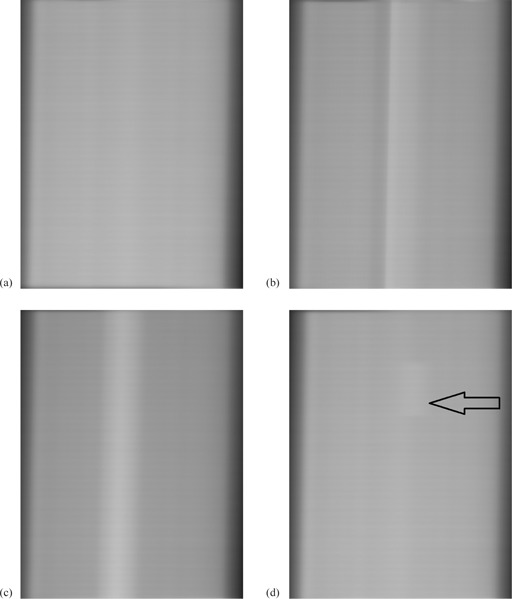
Typical integrated images: (a) delivery without error, (b) 9° gantry position error at one control point, (c) 20 MU dose error at one control point, and (d) 2 mm position error in eight MLC leaves at one control point (arrowed). All images are normalized to an open‐field image and the errors are at gantry angle 90°.

**Figure 5:**
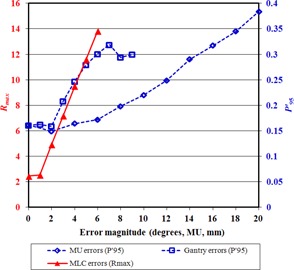
The effect of error magnitude on the parameters used for detection. The horizontal axis refers to degrees in the case of gantry errors, MU in the case of MU errors, and mm in the case of MLC errors.

In the absence of simulated errors, the integrated images are almost uniform, although some features are visible, due to inertial effects in the VMAT delivery (Fig. 4(a)). With simulated delivery errors at gantry 90°, irregular patterns appear in the integrated portal images (Figs. 4(b) to 4(d)). With no delivery error, the $P_{95}^{\prime }$ is 0.15 and $R_{\text{max}}$ is 3.0. With a 4° gantry position error at one control point and an 8 MU dose error at one control point, $P_{95}^{\prime }$ increases to 0.25 (67% increase) and 0.20 (33% increase), respectively. With a 2 mm MLC leaf position error in eight leaves at one control point, $R_{\text{max}}$ increases to 5.1 (increase of 70%). Figure 5 shows the variation of $P_{95}^{\prime }$ and $R_{\text{max}}$ as a function of the error magnitude. From this graph, errors of 3° in gantry angle, 8 MU in dose, and 2 mm in MLC leaf position are expected to be detectable.

**Figure 6:**
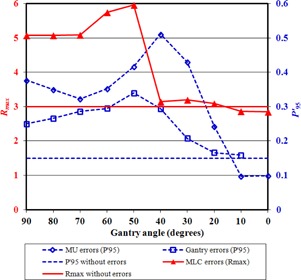
The effect of gantry angle on the parameters used for detection.

The method is more sensitive to errors at gantry angle 90°/270° than at 0°/180° due to the geometry of the test. Errors that occur farther from gantry angle 90°/270° are more difficult to distinguish than at other angles, as the error pattern is superimposed on the edge of the overall integrated image where the intensity is falling off. Due to the sinusoidal motion of the beam aperture across the portal imager and back, most of the aperture motion occurs as the gantry passes though 90°/270° and, for a disproportionately high length of time, the beam aperture irradiates the edge of the intensity pattern. This edge of the intensity pattern is not perfectly sharp, because an aperture sweeping in one direction and then reversing makes an intensity gradient of width equal to the aperture width. An error occurring in this gradient region is difficult to detect. Also, the dose rate is chosen to be proportional to the distance moved by the beam aperture between control points, so as to maintain a uniform portal image without excessive irradiation of the extremities of the intensity pattern. A consequence of this is that errors at gantry angle 0°/180° have less impact than those at 90°/270°, simply because less fluence is delivered per control point at 0°/180°. Figure 6 therefore shows the error detectability as a function of the gantry angle at which the error occurs. For gantry and MU errors, $P_{95}^{\prime }$ remains well above the value for no error from gantry angle 90° to 30°. For MLC errors, which are more subtle, $R_{\text{max}}$ returns to the value for no error at gantry angle 40°. This is because at angles lower than this, the abnormal part of the image is located at the edge of the overall integrated image, making its detection difficult. It is clear from Fig. 6 that there is no loss of sensitivity though from 90° to 50°, so that the sensitivities measured for errors at gantry 90° would be obtained for the whole arc if the test were repeated with the rod offset vertically from the isocenter instead of horizontally.

**Figure 7:**
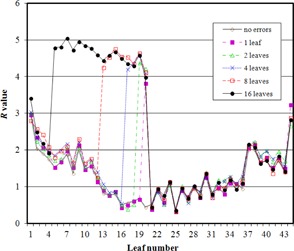
The R‐value for each of the 44 leaves visible in one image, with MLC errors of 2 mm at one control point introduced into various numbers of leaves.

Figure 7 shows the R‐value for each profile with increasing numbers of leaves affected. Because of the use of median and percentile‐based statistics, the number of atypical leaves does not significantly affect the detectability.

The method is also sensitive to positioning of the steel rod (data not shown). A position error of 1 mm shows up in cine images as a movement of the shadow of the rod first to one side then to the other as the gantry rotates, which is unmistakably due to positioning error rather than delivery error. This sensitivity to position is advantageous as the QC procedure inherently tests for the accuracy of the laser alignment system. In practice it is found that the rod can be sufficiently accurately placed using the lasers as to give reproducible results, so long as the lasers are properly positioned.

Considering the value of $P_{95}^{\prime }$ in the absence of errors and with the errors described above, a threshold value of 0.2 is appropriate. This is expected to catch errors affecting all MLC leaves and is effective at the majority of gantry angles. Similarly, a threshold of $R_{\text{max}}$ of 4.0 is appropriate and is expected to detect MLC errors for around half of the gantry rotation. If a greater detection sensitivity is required for this kind of error, the test can be repeated with the geometry rotated by 90°.

**Figure 8:**
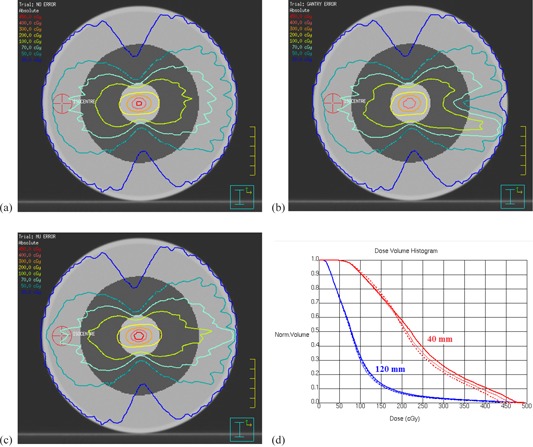
Potential clinical impact of delivery errors: (a) dose distribution without error, (b) dose distribution for a 9° gantry angle error at 90°, (c) dose distribution for a 20 MU dose error at 90°, (d) dose‐volume histograms for the central 40 mm and 120 mm of the cylindrical phantom without error (thin solid lines), with a 9° gantry angle error at 90° (thick dotted lines), and a 20 MU dose error at 90° (thick solid lines). The phantom is homogeneous, and the central 40 mm and 120 mm are shown in grayscale in (a) to (c).

The planning system calculations show that the errors described above are clinically small (Fig. 8). The largest dosimetric errors are seen when the gantry is held back by 9° at gantry 90° and when the monitor units are increased by 20 MU at gantry 90°, with greater impact on the smaller central volume than on the larger volume, while the MLC errors have negligible impact (not shown in Fig. 8 for clarity). These treatment planning calculations show that the errors described are of limited clinical impact, so the method has the power to detect errors before they affect patient treatments. The Delta^4^ verification results are shown in Table 1. It is clear that none of the errors considered have a significant impact on the percentage of measurements passing the gamma criterion, thereby confirming that the synchronization test is able to detect subclinical errors.

**Table 1:** Results of plan verification using a commercial diode array phantom. The table shows the percentage of measurements with gamma (3% and 3 mm) of less than unity when comparing against either the planned dose for the case of no error, or against the measured dose for the case of no error

*Error Type*	Γ *(3% / 3 mm) vs. Planned Dose*	Γ *(3% / 3 mm) vs. Measured Dose*
No error	99.8	‐
4° gantry error	99.5	99.9
9° gantry error	96.2	99.4
8 MU dose error	100.0	100.0
20 MU dose error	99.3	97.2
2 mm MLC error	99.8	100.0
5 mm MLC error	99.8	100.0

### B. Clinical application

Table 2 shows the result of applying the test to five clinical accelerators. Very few error features are visible in the images, with the exception of an MLC calibration error affecting one leaf at all control points on Accelerator 5. Consequently, the mean error detection parameters are below their respective thresholds. The standard deviations of the error metrics vary slightly between accelerators, reflecting consistency of delivery and measurement. In Accelerator 4, the standard deviations of both $P_{95}^{\prime }$ and $R_{\text{max}}$ are low, indicating reproducible delivery, and these standard deviations therefore represent, as close as it is possible to estimate, the inherent uncertainty associated with the test itself. The effect seen on the images for Accelerator 5 has prompted further investigation (by means of the cine images of this test) and it has been found that on this MLC, the ACAL calibration software (Elekta AB) has set the minor gain parameter for one leaf to be 2056, different from the 2048 of the adjacent leaves. While this difference is imperceptible on normal MLC calibration images, it shows clearly on the VMAT synchronization test. Once the minor gain for the anomalous leaf is adjusted to 2048, the value of $R_{\text{max}}$ reduces from 4.4 to 2.6. This is, therefore, a clear demonstration of the sensitivity and utility of the VMAT synchronization test.

**Table 2:** Results of VMAT QC on five linear accelerators. The table shows the mean (±1SD) of the quality parameters over the available images

*Accelerator*	*MLC Type*	$P_{95}^{\prime }$ *(tolerance 0.2)*	$R_{\text{max}}$ *(tolerance 4.0)*
1	MLCi	0.12±0.006	3.0±0.9
2	MLCi	0.18±0.087	3.7±1.0
3	Beam Modulator	0.14±0.005	3.7±0.8
4	Beam Modulator	0.14±0.013	3.3±0.2
5	Beam Modulator	0.13±0.006	4.4±0.2 before adjustment; 2.6 after adjustment

## IV. DISCUSSION

There are several advantages to this method of VMAT QC. The first is that the method is based on a geometrical foundation, so that there is a clear definition of a normal VMAT delivery. Several other methods^20^, ^21^ use a sliding window which moves during delivery of an arc and this approach is similar to that used in our paper. However, in the absence of something specific with which to synchronize (in this case the steel bar), these other methods require a normal dose distribution to be measured and used as a standard. In contrast, the present method can be widely applied across a number of accelerators, and in all cases a normal delivery gives a uniform image and an abnormal delivery some sort of excursion from a uniform image. The open reference images assist with accurate numerical analysis, but are not an absolute prerequisite for the method. Because the method is measuring integrated beam fluence, as opposed to absolute dose, the method is expected to be independent of photon energy, although this has not at present been verified. A fully dosimetric characterization of the portal imager under buildup is also not required. Absolute dosimetry during the VMAT arc should be measured using other methods. However, it may be possible to incorporate a narrow cylindrical water‐equivalent phantom around the steel bar, so that the method can be used, in conjunction with a theoretically predicted portal image, as an absolute dose check, similar to current methods of portal dosimetry.^(25)^

A further advantage of the method described in this paper is that the method requires very little equipment for its operation, which is a practical advantage during a busy monthly quality control session. In our case, the laser QC board, to which the steel rod is attached, can be used for other parts of the QC session, as well. The test is fast to perform, and the images can be analyzed in as much or as little detail as is required. The tests are designed so that a significant delivery error can be visualized on the images immediately, and this is also important practically. Numerical analysis then provides a more precise result for consideration against tolerances and for recording purposes. The time taken for the VMAT QC session is approximately 5 min for setup of the laser QC board and bar, 2 min to deliver the first arc, and 2 min to deliver the second arc. This may then be repeated for the orthogonal test, taking 18 min in total. The visual analysis takes 2 min, so that a good indication of the accelerator performance can be obtained after 20 min. Off‐line numerical analysis then takes a further 20 min. In the event of a significant error being observed, a further 20 min for acquisition of cine images is required. Thus, brief results are obtained after 20 min, and complete results in less than 60 min.

A similar geometrical approach has been taken by Wang et al.^(17)^ using a cylindrical array phantom. Their method is designed to irradiate specific diodes in the phantom with specific control points of the arc. The method is able to detect MLC leaf errors of the order of 0.5 mm, while gantry angle and dose rate are determined every 50 ms, although a calibration between diode response and spatial error is needed for each leaf at each control point to obtain this information. The present method is less sensitive than this, but still sensitive enough to be able to detect subclinical errors. The imaging panels used for this study are routinely used for *in vivo* portal dosimetry,^25^, ^26^ so their intrinsic calibration, homogeneity, and noise level are known to be stable and reproducible to within several percent.^(27)^ Furthermore, the homogeneity of the images is improved by the use of the open reference image. However, some uncertainties remain. These are primarily image noise, image resolution, image homogeneity, image distortion, rod positioning, and panel sag, of which the latter two are likely to be the most significant. These uncertainties are predominantly spatial, which by means of the moving aperture, transfer into intensity uncertainties. Even in well‐commissioned properly functioning accelerators, there is some variability from accelerator to accelerator in MLC calibration and dose rate, and the performance statistics in the absence of clinically significant errors are therefore not zero. However, Table 2 indicates that the normal levels and intrinsic test uncertainties are $P_{95}^{\prime }$ = 0.15±0.01(1SD) and $R_{\text{max}}=3.0\pm 0.2(1\;\text{SD})$. The tolerance levels of $P_{95}^{\prime }=0.2$ and $R_{\text{max}}=4.0$ are designed to be high enough that they are not exceeded by normally functioning accelerators, but low enough that they catch any errors before they become clinically noticeable. The results of the Delta^4^ analysis show that, even when the thresholds are exceeded, the errors are clinically indistinguishable.

It is possible that the images obtained in the absence of errors could be made more consistent by introducing a flex map into the procedure so as to overcome panel sag.^(28)^ The panel sag on the imaging panels used in this study, measured by determining the offset of measured images against their position predicted from the accelerator readout of MLC leaf positions, is in the order of 5 mm peak‐peak (in‐plane) and 3 mm peak‐peak (cross‐plane).^(29)^ Correcting for this might enable the tolerance level for the method to be lowered, thereby increasing the sensitivity of the method to errors. However, the results of this study indicate that panel inertia is not a significant effect.

The methods of Liu et al.^(19)^ and Wang et al.^(17)^ use cine acquisition of images and dose, enabling the course of the beam delivery to be analyzed in detail. This is an option for our method also, but from a practical perspective, it is valuable to have a single acquired image which reflects the delivery accuracy, and this has therefore been the focus of our study. In the event of a discrepancy in the overall image, cine images can be acquired and analyzed, as was in fact done when a discrepancy was found and corrected on Accelerator 5. Due to the short acquisition time for each cine image, the cine results are prone to artefact and are, therefore, only analyzed visually. However, this is sufficient to identify the position of the MLC leaves with respect to the bar to within ± 0.5 mm. The cine images are sufficient to locate multiple errors occurring at various locations in the arc. However, multiple errors occurring simultaneously are difficult to identify with this method and such errors, once they have been demonstrated with the present method, would be best investigated by multiple simple tests, such as sliding window tests at a range of gantry angles, or by means of a dosimetric phantom.

## V. CONCLUSIONS

The proposed method allows the performance of VMAT delivery to be assessed conveniently and rapidly using one or two arcs, and is suitable for inclusion in a monthly accelerator QC program. The test is able to detect errors in the delivery of individual control points, with the possibility of using movie images to further investigate suspicious image features. The method is sensitive to small delivery errors in gantry position, dose rate, and MLC leaf position, with greater sensitivity being shown at gantry angles 90° and 270° due to the geometry of the test. This method is now in routine clinical use as part of the monthly QC tests on each linear accelerator performing VMAT at our center.

## ACKNOWLEDGMENTS

We are grateful for NHS funding to the NIHR Biomedical Research Centre at The Royal Marsden and the ICR. We would also like to thank Elekta AB (Stockholm) for their collaboration on VMAT and Agility. We appreciate the assistance given by the Trust's mechanical and radiotherapy engineers during this project.
